# Sevoflurane preconditioning promotes mesenchymal stem cells to relieve myocardial ischemia/reperfusion injury via TRPC6-induced angiogenesis

**DOI:** 10.1186/s13287-021-02649-3

**Published:** 2021-11-22

**Authors:** Jinting Yang, Lihui Tang, Fengjiang Zhang, Tingting Yang, Ting Lu, Kai Sun, Na Sun, Jinxuan Ren, Min Yan

**Affiliations:** 1 Department of Anesthesiology, The Second Affiliated Hospital, Zhejiang University School of Medicine, Hangzhou, 310009 China; 2 Clinical Skill Training Center, The Second Affiliated Hospital, Zhejiang University School of Medicine, Hangzhou, 310009 China; 3 Department of Anesthesiology, The First Affiliated Hospital of Xi’an Jiaotong University School of Medicine, Xi’an, 710061 China; 4 Clinical Research Center, The Second Affiliated Hospital, Zhejiang University School of Medicine, Hangzhou, 310009 China

**Keywords:** Mesenchymal stem cells, Sevoflurane, Myocardial ischemia/reperfusion injury, Angiogenesis, Transient receptor potential canonical channel 6

## Abstract

**Background:**

Ischemic heart diseases is one of the leading causes of death worldwide. Although revascularization timely is an effective therapeutic intervention to salvage the ischemic myocardium, reperfusion itself causes additional myocardial injury called ischemia/reperfusion (I/R) injury. Bone marrow-derived mesenchymal stem cells (MSCs) is one of the promising cells to alleviate ischemic myocardial injury. However, this cell therapy is limited by poor MSCs survival after transplantation. Here, we investigated whether sevoflurane preconditioning could promote MSCs to attenuate myocardial I/R injury via transient receptor potential canonical channel 6 (TRPC6)-induced angiogenesis.

**Methods:**

The anti-apoptotic effect of sevoflurane preconditioning on MSCs was determined by Annexin V-FITC/propidium iodide staining. TRPC6, hypoxia-inducible factor-1α (HIF-1α), Chemokine receptor 4 (CXCR4) and vascular endothelial growth factor (VEGF) protein expressions and VEGF release from MSCs were determined after hypoxia and reoxygenation (H/R). Small interfering RNA (siRNA) was used to knock down *TRPC6* gene expression in MSCs. The angiogenesis of human umbilical vein endothelial cells (HUVECs) co-cultured with MSCs was determined by Matrigel tube formation. Myocardial I/R mouse model was induced by occluding left anterior descending coronary artery for 30 min and then reperfusion. MSCs or sevoflurane preconditioned MSCs were injected around the ligature border zone 5 min before reperfusion. Left ventricle systolic function, infarction size, serum LDH, cTnI and inflammatory cytokines were determined after reperfusion.

**Results:**

Sevoflurane preconditioning up-regulated TRPC6, HIF-1α, CXCR4 and VEGF expressions in MSCs and VEGF release from MSCs under H/R, which were reversed by knockdown of *TRPC6* gene using siRNA in MSCs. Furthermore, sevoflurane preconditioning promoted the angiogenic and anti-inflammatory effect of HUVECs co-cultured with MSCs. Sevoflurane preconditioned MSCs improved left ventricle systolic function and alleviated myocardial infarction and inflammation in mice subjected to I/R insult.

**Conclusion:**

The current findings reveal that sevoflurane preconditioned MSCs boost angiogenesis in HUVECs subjected to H/R insult and attenuate myocardial I/R injury, which may be mediated by TRPC6 up-regulated HIF-1α, CXCR4 and VEGF.

**Supplementary Information:**

The online version contains supplementary material available at 10.1186/s13287-021-02649-3.

## Background

In China, more than 17 million people are suffering from cardiovascular diseases, and mortality rate due to coronary heart diseases is more than 1/1000 in 2014 [1]. Ischemic heart diseases is one of the leading causes of death worldwide. Timely revascularization is an effective therapeutic intervention to salvage the ischemic myocardium, but reperfusion itself causes additional myocardial injury called ischemia/reperfusion (I/R) injury. There is still no effective therapy to prevent myocardial I/R injury. Bone marrow-derived mesenchymal stem cells (MSCs) are non-hematopoietic subpopulation cells with differentiation potential into various tissues, which can be isolated from bone marrow, adipose, synovial tissue, lung, umbilical cord blood, peripheral blood and olfactory bulbs [2]. MSCs are the promising cell therapeutic approach due to its self-renewal, homing potential, paracrine actions and vascularization [3]. However, the benefit of MSCs therapy is limited by the poor cell survival after transplantation and innovative approaches are required to improve the viability and therapeutic potency of MSCs.

A growing body of evidence demonstrates that sevoflurane, a volatile anesthetic, preconditioning or postconditioning protects organs against I/R injury and other insults by anti-inflammatory response and anti-oxidative effect [4–7]. We previously found that sevoflurane preconditioning activates reperfusion injury salvage kinase pathways to improve left ventricle systolic function and reduce infarct size [8]. What’s more, sevoflurane preconditioning up-regulates hypoxia-inducible factor-1α (HIF-1α) and vascular endothelial growth factor (VEGF) to improve MSCs viability and migration against hypoxia and serum deprivation [9]. These results indicate that sevoflurane preconditioning might be an innovative approach to enhance the cardioprotective effect of MSCs against I/R injury.

Transient receptor potential (TRP) is a superfamily of cation channels distributed throughout the mammalian body. TRP channels act a pivotal part in regulating intracellular Ca^2+^ concentration and subcellular Ca^2+^ signaling in endothelial cells, which directly influences endothelium-mediated relaxation and angiogenesis [10]. TRP canonical channel 6 (TRPC6), one of TRP family, regulates intracellular Ca^2+^ and Na^+^ influx at the fifth and sixth transmembrane domains of a pore [10]. Intracellular Ca^2+^ signals are essential to modulate proliferation, motility and angiogenesis of vascular endothelial cells [11], and the involvement of TRPC6 in this Ca^2+^ influx response has been characterized. In rat retinal Müller cells, TRPC6 silencing decreases VEGF level [12]. These results indicate that TRPC6 is essential to take part in angiogenesis. However, whether sevoflurane preconditioned MSCs promotes human umbilical vein endothelial cells (HUVECs) angiogenesis under H/R through TRPC6-related pathway remains unclear. Here, we investigated the possible mechanism by which sevoflurane preconditioned MSCs promote angiogenesis in HUVECs under H/R and the potential therapeutic role of sevoflurane preconditioned MSCs against myocardial I/R injury in vivo.

## Methods

### Animal and cells

C57BL/6 mice (8–10 weeks old) were purchased from Model Animal Research Center of Nanjing University. They were kept in a controlled room (22 °C ± 1 °C and a 12 h/12 h light/dark cycle) and fed on standard pellet chow and water ad libitum. All animal experiments were in compliance with the National Institute of Health Guide for the Care and Use of Laboratory Animals and were approved by the Ethics Committee for Animal Experimentation of 2nd Affiliated Hospital, Zhejiang University School of Medicine (Hangzhou, China).

Primary bone marrow-derived MSCs were purchased from the Stem Cell Bank, Chinese Academy of Sciences (Shanghai, China). After 3–5 passages, MSCs were cultured in MEM-α medium including 10% fetal bovine serum (FBS) and 1% penicillin–streptomycin at 37℃. Primary HUVECs were a gift from Professor Jianan Wang (Zhejiang University School of Medicine, Hangzhou, China). HUVECs were incubated in low glucose DMEM supplemented with 10% FBS and antibiotics (1% penicillin–streptomycin) for 4–8 passages. All medium and FBS were purchased from Gibco (Grand Island, NY, USA).

### Sevoflurane preconditioning

MSCs were plated at 1 × 10^5^ cells/cm^2^ and placed in an airtight and humidified chamber. MSCs were preconditioned with sevoflurane according to the previous report [9]. The chamber was flushed with the mixture gas (21% O_2_, 5% CO_2_) for 5 min and balanced with 2% sevoflurane for 2 h. The mixture gas and sevoflurane were supplied by an in-line anesthetic vaporizer (Drager Vamos, Lübeck, Germany). Concentration of sevoflurane was real-time monitored by an anesthetic analyzer (Drager Vamos, Lübeck, Germany). Control cells were placed under 21% O_2_ and 5% CO_2_.

### Hypoxia and reoxygenation

MSCs were seeded at 1 × 10^5^ cells/cm^2^ in the complete culture medium. They were placed under normoxic incubator containing 21% O_2_ and 5% CO_2_ or hypoxia incubator with 0.5% O_2_ and 5% CO_2_ using the ProOXC21 system (BioSpherix, Redfield, NY, USA) for 12 h at 37 ℃ as previously described [13] and then transferred to normoxia incubator for 2 h as reoxygenation (H/R).

### MSCs apoptosis assay

After H/R, the apoptosis of MSCs with or without sevoflurane preconditioning was measured by FITC-Annexin V/propidium iodide (AV/PI) apoptosis detection kit (BD Biosciences, San Diego, CA, USA) as previously described [14]. Cell apoptosis was detected and analyzed by BD FACS Calibur Flow Cytometer.

### Transfection of siRNA

As previously described [15], MSCs were cultured in six-well plate at 5 × 10^4^ cells/cm^2^ for 24 h and transfected with TRPC6 siRNA constructed from GenePharma (Shanghai, China) in the presence of Lipofectamine 2000 (Invitrogen, USA). After 48 h transfection, MSCs were pretreated with 2% sevoflurane for 2 h and then were exposed to H/R (12 h/2 h). After that, MSCs and the supernatant were collected.

### Tube formation of HUVECs co-cultured with MSCs

After MSCs (1 × 10^5^ cells/cm^2^) were seeded in an insert of transwell in 24-well plates in the normoxic incubator for 24 h, they were preconditioned by sevoflurane. A Matrigel tube-formation assay was adopted to determine angiogenesis in vitro as previously described [16]. HUVECs (2.5 × 10^4^ cells/cm^2^) were plated into the lower transwell chamber with low glucose DMEM in 24-well plates pre-coated with growth factor-reduced Matrigel (BD Biosciences). MSCs and HUVECs were co-cultured in two chambers separated by a polycarbonate membrane with a 0.4 μm pore exposed to normoxia or hypoxia circumstance. After 4–6 h, the total number of interbranches (tube formation) in each well was observed and quantified by counting five fields (original magnification, × 100) using Image-Pro Plus 6.0 (Media Cybernetics, USA). The supernatant was collected after co-culture.

### Myocardial I/R mouse model

The myocardial I/R mouse model was established as previously described [17]. The mouse was anesthetized by pentobarbital sodium (60 mg/kg, i.p.) and was ventilated with the tracheal intubation connected to a mechanical ventilator. Left anterior descending (LAD) coronary artery was occluded using a 7–0 nylon suture. The immediate color changes of the vessel and cardiac tissue from distal to the ligature confirmed that LAD was blocked successfully. The mouse heart was exposed to 30 min ischemia and reperfusion forever. Mice in sham group underwent thoracotomy but without LAD ligation. When the heart was exposed to ischemia for 25 min, PBS, MSCs or sevoflurane preconditioned MSCs (2 × 10^5^ cells total) in 30 μl were injected at 5 sites around the ligature border zone.

### Echocardiography

After 3 days’ and 7 days’ reperfusion, the left ventricular (LV) function of mice was analyzed using transthoracic echocardiography Vevo 2100 system (VisualSonics, Toronto, Canada) as previously described [17]. Briefly, isoflurane (0.5–1% in oxygen) was used to anesthetize mice in the supine position. M-mode tracing images at short axis were adopted to measure LV wall thickness and chamber diameters. The in-built software package of echocardiography was used to calculate LV end-diastolic diameter (LVEDD), end-systolic diameter (LVESD), percentage of ejection fraction (EF) and fraction shortening (FS).

### Determination of myocardial infarct size

A double staining of Evans blue and TTC was used to determine myocardial infarct size as previously reported [17]. Briefly, the LAD was re-occluded at the same location and 1% Evans blue was retrogradely injected into coronary arteries to stain the myocardium except for the area at risk (AAR) at 3 d after I/R. The heart was cut into 2-mm slices and incubated with 1% TTC (37 °C) for 15 min. The volume of total slices, AAR and infarction size were determined using Image-Pro Plus 6.0. AAR was calculated as a percentage of the entire ventricular area, and infarct size was expressed as percentage of AAR.

### Determination of LDH, cTnI, VEGF, IL-1β, TNF-α and IL-10

LDH level in the supernatant of HUVECs and in the serum of mice subjected to I/R injury was measured by a commercial kit (Nanjing Jiancheng Bioengineering Institute, Nanjing, China). VEGF, IL-1β, TNF-α and IL-10 levels in the supernatant of MSCs and cTnI, IL-1β, TNF-α and IL-10 levels in the serum of mice were determined by ELISA kits (Elabscience, Wuhan, China).

### Western blot

At the end of H/R, MSCs were collected. As we described elsewhere [18, 19], lysates were separated on 10% SDS-PAGE gels and then transferred to polyvinylidene fluoride membranes. The membranes were incubated with the following primary antibodies: TRPC6 (1:500, Abcam, Cambridge, MA, USA), HIF-1α (1:500, Abcam, Cambridge, MA, USA), CXCR4 (1:1000, Abcam, Cambridge, MA, USA), VEGF (1:1000, Abcam, Cambridge, MA, USA) and GAPDH (1:5000, Beyotime Biotechnology, Shanghai, China) overnight at 4 °C. After washing, membranes were incubated with horse radish peroxidase-conjugated secondary antibodies for 1 h. Blotting bands were visualized using an ECL kit and captured by the BIO-RAD ChemiDoc Touch Imaging System.

### Quantitative real-time PCR

As we previously described [18, 19], total RNA was extracted from MSCs using Trizol reagent. RNA was reversely transcribed into complementary DNA (cDNA) using the PrimeScript RT reagent Kit (Takara Bio Inc, Dalian, China). The quantitative RT-PCR (qRT-PCR) was performed with TB Green premix Ex Taq II (Takara Bio Inc, Dalian, China). The specific Primers and according sequences are displayed as follows: β-actin, forward 5′-CACTATCGGCAATGAGCGGTTCC-3′ and reverse 5′-CAGCACTGTGTTGGCATAGAGGTC-3′; TRPC6 forward 5′-TTCCTCGTGGTCCTCGCTGTC-3′ and reverse 5′-GAAGGAGGCCGCATGTGCTAC-3′. Results were normalized to β-actin mRNA level and expressed as 2^−ΔΔCt^.

### Statistical analysis

GraphPad Prism 5.0 software (GraphPad Prism, San Diego, CA, USA) was used to analyze data expressed as Mean ± SEM. Differences were made using one-way ANOVA with Newman–Keuls test among multiple groups or Student’s *t* test between two groups. A value of *P* < 0.05 was considered statistically significant.

## Results

### Sevoflurane preconditioning relieves apoptosis and up-regulates TRPC6 and its target VEGF in MSCs under H/R

**Fig. 1:**
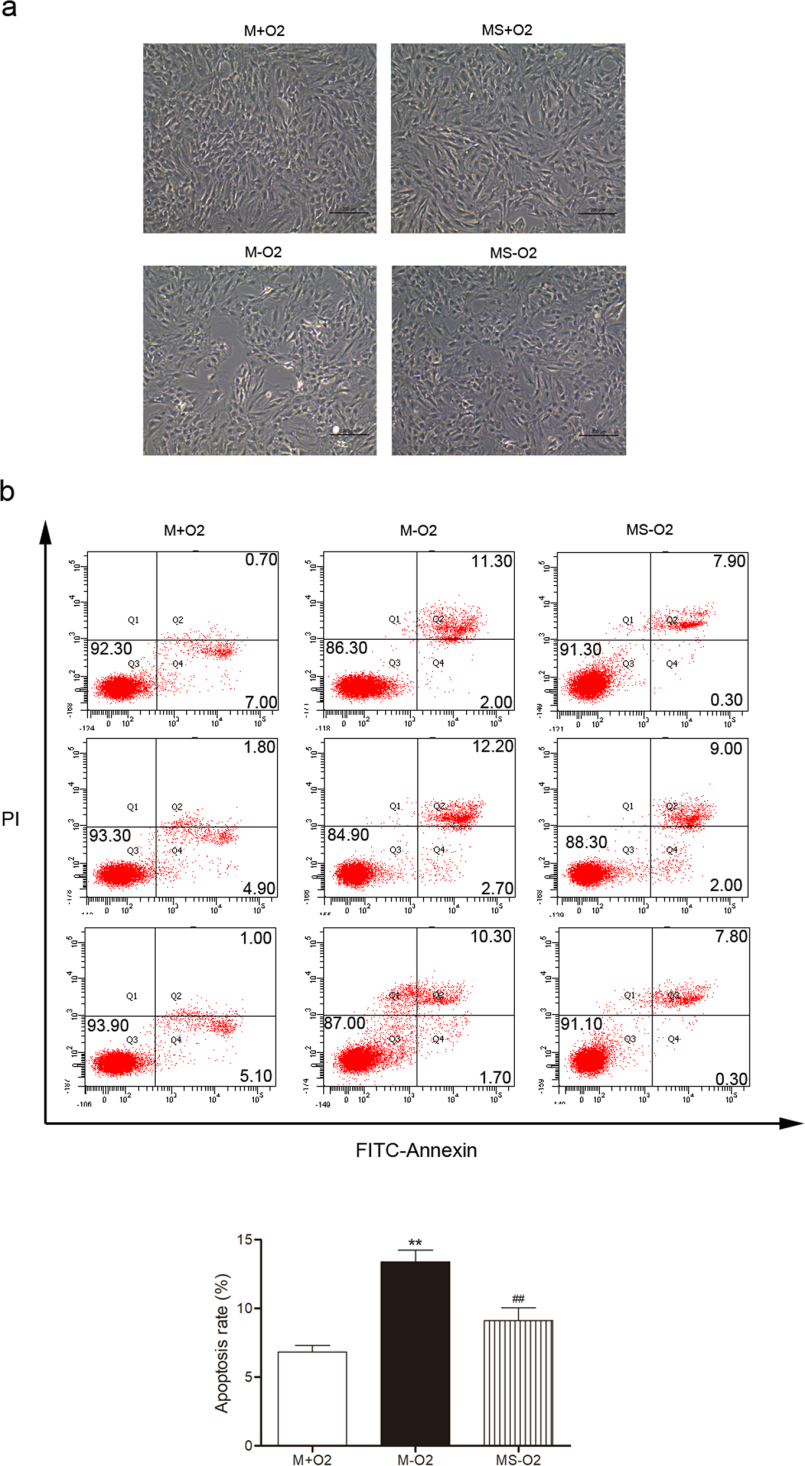
Sevoflurane preconditioning attenuated cellular apoptosis in MSCs under hypoxia and reoxygenation. The morphological alterations of MSCs with or without sevoflurane preconditioning under 12 h hypoxia and 2 h reoxygenation (H/R) (**a**). M + O_2_, MSCs under normoxia; MS + O_2_, sevoflurane preconditioned MSCs under normoxia; M-O_2_, MSCs under H/R; MS-O_2_, sevoflurane preconditioned MSCs under H/R. Scale bar = 200 μm. The effect of sevoflurane preconditioning on apoptosis in MSCs under H/R (**b**). Data are shown as Mean ± SEM, *n* = 3 per group, ^**^P < 0.01 versus M + O_2_, ^##^*P* < 0.01 versus M-O_2_

**Fig. 2:**
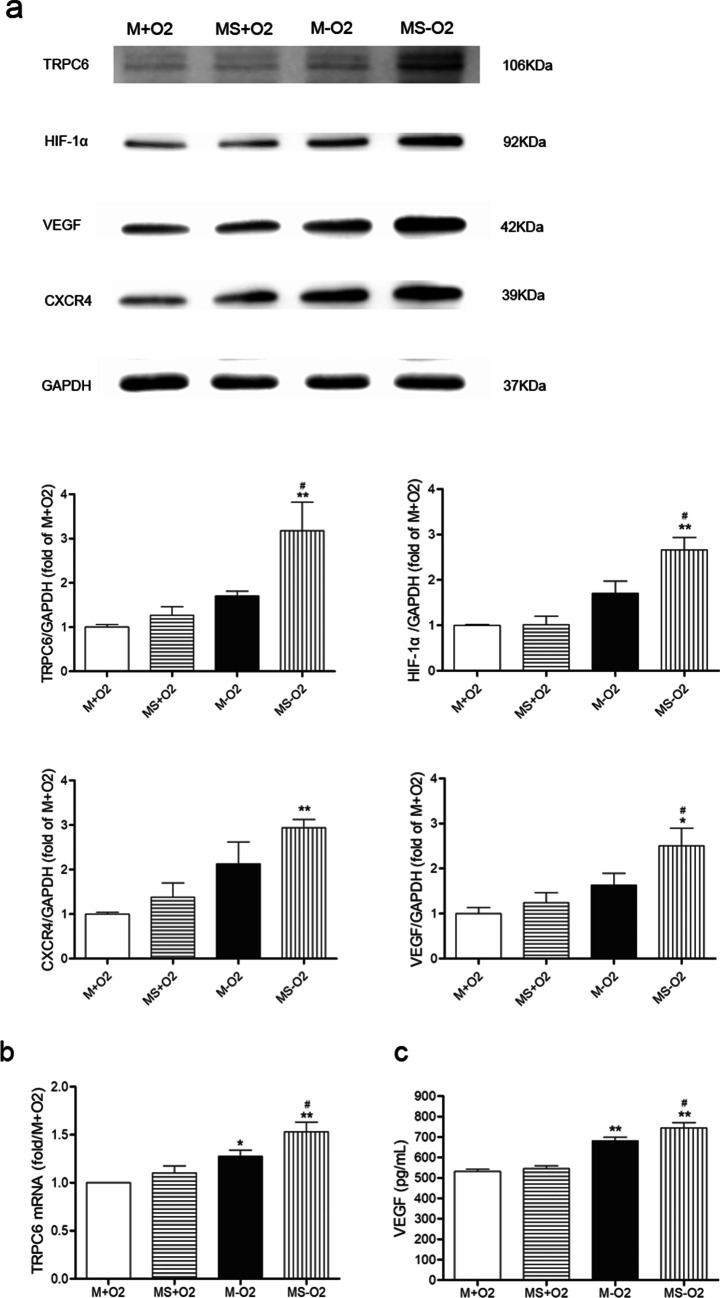
Sevoflurane preconditioning up-regulated TRPC6, HIF-1α, VEGF and CXCR4 in MSCs under H/R. Protein expressions of TRPC6, HIF-1α, VEGF and CXCR4 in MSCs under H/R (**a**). mRNA expression of TRPC6 in MSCs under H/R (**b**). VEGF level detected by ELISA assay in the supernatant of MSCs under H/R (**c**). Data are shown as Mean ± SEM, *n* = 3 per group (**a**), *n* = 5 per group (b and c). ^*^P < 0.05, ^**^P < 0.01 versus M + O_2_; ^#^*P* < 0.05 versus M-O_2_

**Fig. 3:**
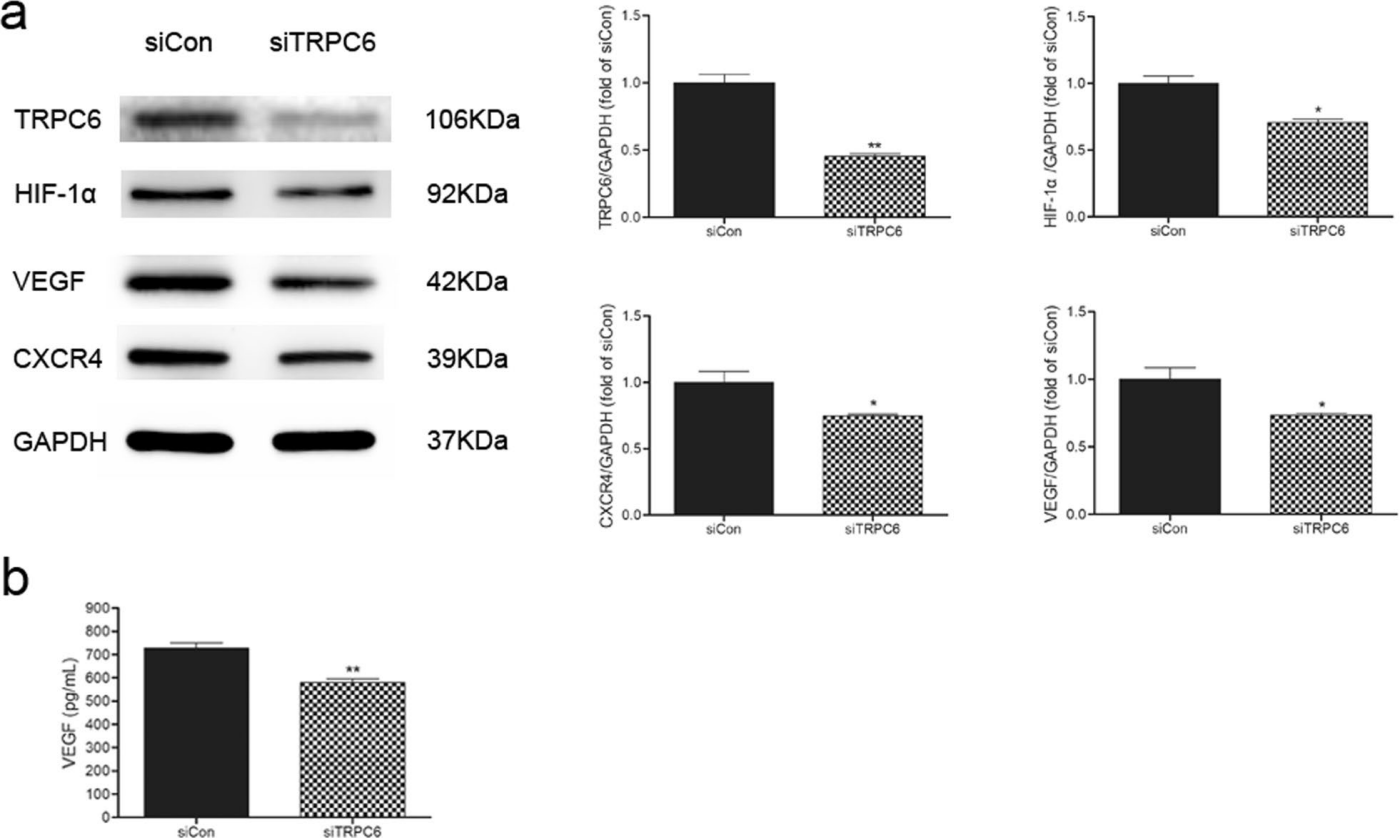
VEGF expression was decreased by TRPC6-knockdown in sevoflurane preconditioned MSCs under H/R. Representative images and quantification of Western Blotting to access protein expression of TRPC6, HIF-1α, VEGF and CXCR4 in all groups (**a**). VEGF level in the supernatant was measured by ELISA kits (**b**). Data are shown as Mean ± SEM, *n* = 3 per group. ^*^P < 0.05, ^**^P < 0.01 versus siCon (negative siRNA treated MSCs under H/R), siTRPC6 groups (TRPC6-siRNA treated MSCs under H/R)

As shown in Fig. 1a, cell swelling and membrane rupture were markedly increased in the MSCs subjected to H/R, which were significantly alleviated by sevoflurane preconditioning. The apoptosis of MSCs was increased in the H/R group compared with that in the normoxic group, which were markedly inhibited by sevoflurane preconditioning (Fig. 1b). We also found that sevoflurane preconditioning significantly attenuated the down-regulation of anti-apoptotic protein Bcl-2 (0.79 ± 0.09 vs 0.45 ± 0.02) and the up-regulation of pro-apoptotic protein Bax (1.46 ± 0.10 vs 1.79 ± 0.08) in MSCs after H/R insult (Additional file 1: Fig. S1), which at least indicates that sevoflurane preconditioning benefits the survival of MSCs under H/R partly via handling Bcl-2/Bax-related apoptotic pathway. As shown in Fig. 2c, VEGF release was obviously increased in the H/R group compared with that in the normoxic group and further markedly augmented by sevoflurane preconditioning in MSCs under H/R. Furthermore, TRPC6, HIF-1α, CXCR4 and VEGF expressions were markedly up-regulated by sevoflurane preconditioning in MSCs under H/R (Fig. 2a). In consistent with the result of protein expression, TRPC6 mRNA was significantly increased by sevoflurane preconditioning in MSCs under H/R (Fig. 2b). After using TRPC6 siRNA, the down-regulation of TRPC6 was also accompanied by markedly decreased expressions of HIF-1α, CXCR4 and VEGF in sevoflurane preconditioned MSCs under H/R (Fig. 3a). Moreover, the increased VEGF release from sevoflurane preconditioned MSCs under H/R was markedly blocked by TRPC6 siRNA (Fig. 3b).

### Sevoflurane preconditioning promotes the angiogenic effect of MSCs

**Fig. 4:**
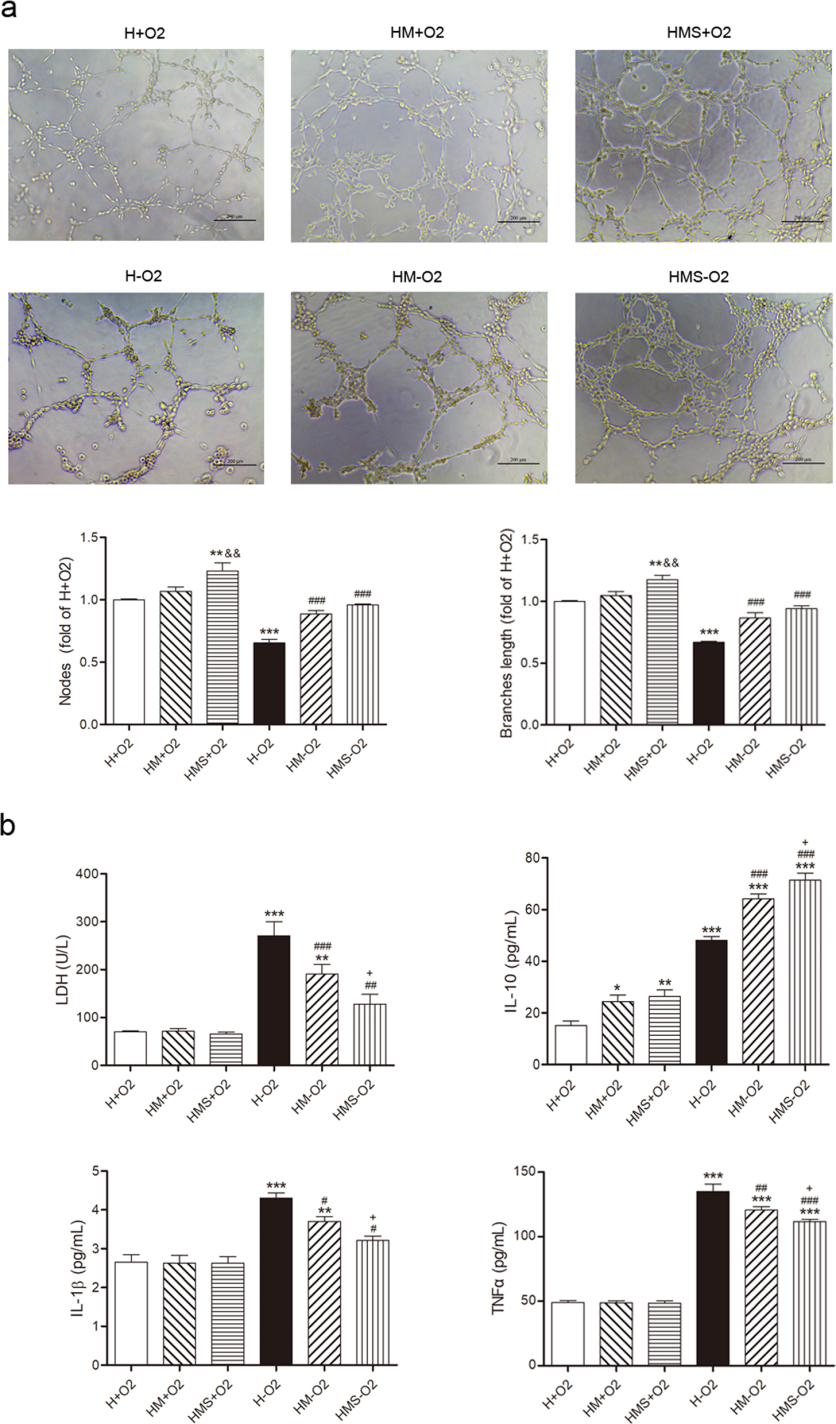
Angiogenic effect of sevoflurane preconditioned MSCs in vitro. Representative images (scale bar = 200 μm) and quantification of nodes and branches length (that indicated tube formation) of HUVECs co-cultured with sevoflurane preconditioned MSCs in Matrigel for 4 or 6 h (**a**). LDH, IL10, IL-1β and TNF-α levels of HUVECs co-cultured with sevoflurane preconditioned MSCs under H/R (**b**). H + O_2_, HUVECs under normoxia; HM + O_2_, HUVECs co-cultured with MSCs under normoxia; HMS + O_2_, HUVECs co-cultured with sevoflurane preconditioned MSCs under normoxia; H-O_2_, HUVECs under H/R; HM-O_2_, HUVECs co-cultured with MSCs under H/R; HMS-O_2_, HUVECs co-cultured with sevoflurane preconditioned MSCs under H/R. Data are shown as Mean ± SEM, *n* = 3 per group. ^*^P < 0.05, ^**^P < 0.01, ^***^P < 0.001 versus H + O_2_; ^&&^*P* < 0.01 versus HM + O_2_; ^#^*P* < 0.05, ^##^*P* < 0.01, ^###^*P* < 0.001 versus H-O_2_; ^+^*P* < 0.05 versus HM-O_2_

Tube formation, an assay indicating angiogenesis, with capillary-like extensions and vascular networks in HUVECs under H/R was both significantly enhanced by both MSCs and sevoflurane preconditioned MSCs (Fig. 4a). In addition, sevoflurane preconditioned MSCs significantly promoted tube formation in HUVECs under normoxia compared with that in MSCs alone group (Fig. 4a). LDH release, a biomarker of cellular membrane rupture, in HUVECs under H/R was significantly increased compared with that in normoxic group (Fig. 4b), which was markedly attenuated by both MSCs and sevoflurane preconditioned MSCs (Fig. 4b). LDH was further reduced by sevoflurane preconditioned MSCs under H/R compared with that in MSCs alone group (Fig. 4b). The increase in pro-inflammatory cytokines IL-1β and TNF-α levels in the supernatant of HUVECs under H/R was markedly attenuated by both MSCs and sevoflurane preconditioned MSCs (Fig. 4b). IL-10 levels were increased in the supernatant of HUVECs under H/R, which were further raised by both MSCs and sevoflurane preconditioned MSCs (Fig. 4b). IL-10 levels were further increased by sevoflurane preconditioned MSCs compared with that in MSCs alone group under H/R (Fig. 4b).

### Sevoflurane preconditioning improves the cardiac protection of MSCs against I/R injury

**Fig. 5:**
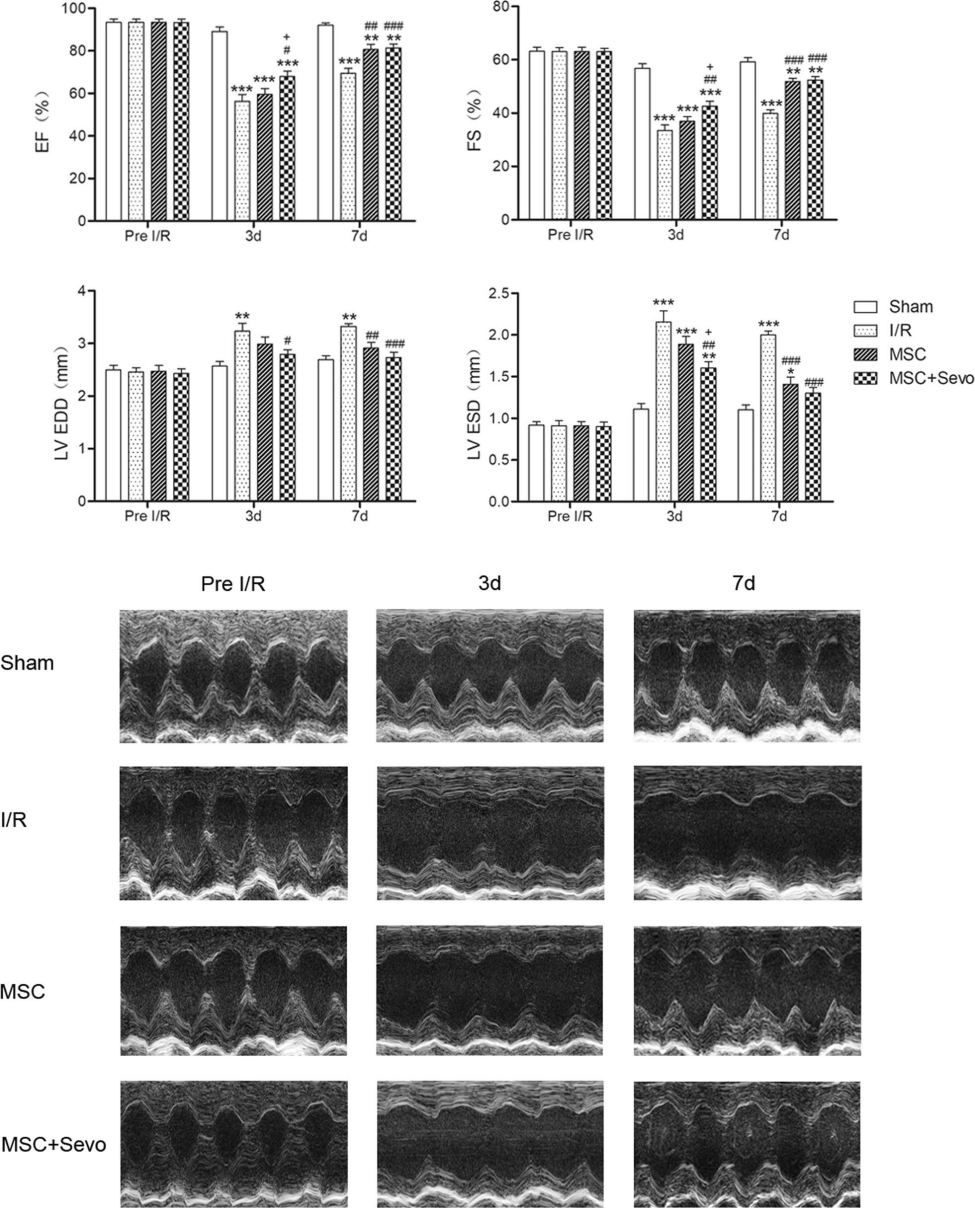
Transplanted sevoflurane preconditioned MSCs improved cardiac function in mice after I/R. Representative images and quantification of echocardiography were shown as before I/R, 3 d and 7 d after I/R. Ejection fraction (EF), fraction shortening (FS), left ventricular end-diastolic diameter (LVEDD), left ventricular end-systolic diameter (LVESD) were obtained to evaluate the cardiac function in each groups. Sham, mice underwent thoracotomy but without LAD ligation; I/R, MSC and MSC + Sevo, mice subjected to ischemia (30 min) and injected with PBS, MSCs or sevoflurane preconditioned MSCs at 5 min before reperfusion. Data are shown as Mean ± SEM, *n* = 7 per group. ^*^P < 0.05, ^**^P < 0.01, ^***^P < 0.001 versus Sham; ^#^*P* < 0.05, ^##^*P* < 0.01, ^###^*P* < 0.001 versus I/R, ^+^*P* < 0.05 versus MSC

As shown in Fig. 5, transplantation of MSCs and sevoflurane preconditioned MSCs significantly augmented systolic function as evidenced by the improvement of EF and FS compared with those in I/R group after reperfusion for 3 d and 7 d. Furthermore, sevoflurane preconditioned MSCs markedly increased EF compared with that in MSCs group after reperfusion for 3 d. As revealed in Fig. 5, LVEDD and LVESD were both markedly increased in I/R group after reperfusion for 3 d and 7 d compared with those in sham group, which were reversed by sevoflurane preconditioned MSCs after reperfusion for 3 d and 7 d. However, there was no significant difference of all echocardiographic parameters between transplantation of sevoflurane preconditioned MSCs and MSCs alone after reperfusion for 7 d. With reperfusion, the contractile function remains depressed due to the “stunned” myocardium and gradually returns to normal after 1 week of reperfusion [20], which accounts for some of the delayed heart functional improvement following reperfusion. To study the cardiac protective effect of sevoflurane preconditioned MSCs, the following assay was determined after reperfusion for 3 d.

**Fig. 6:**
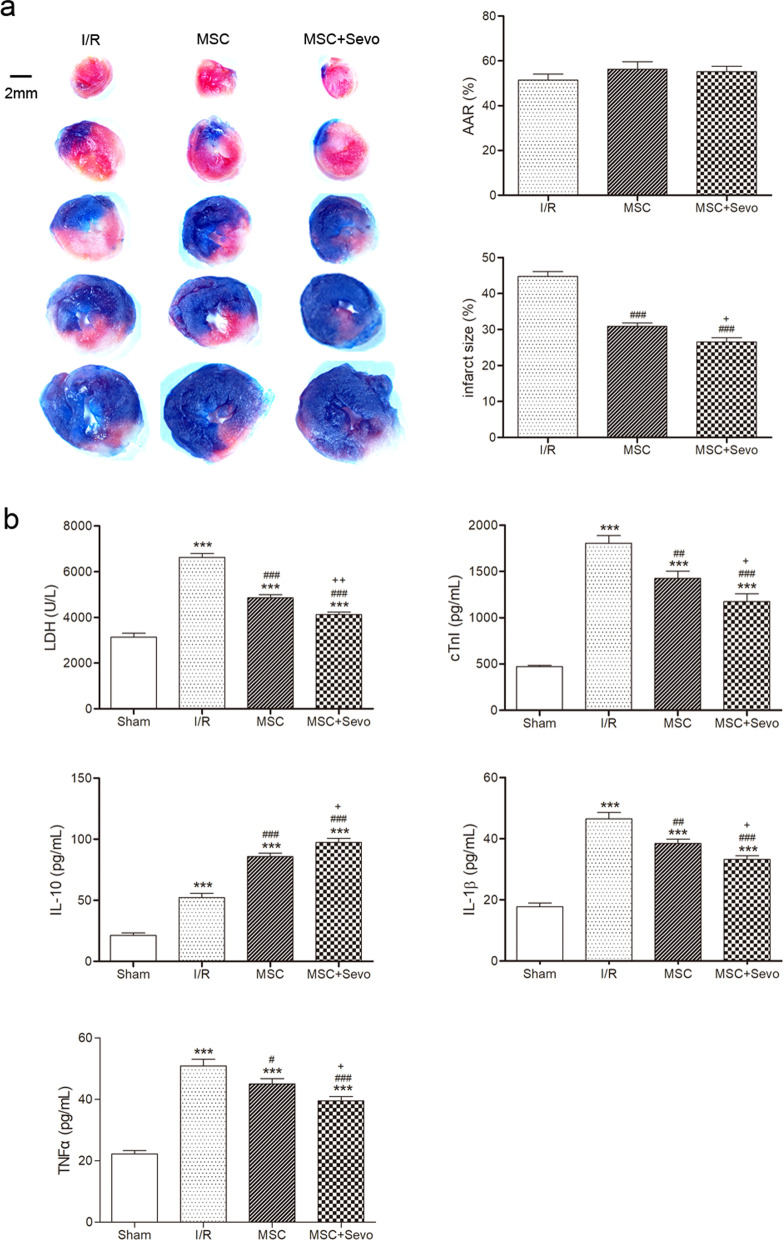
Transplanted sevoflurane preconditioned MSCs reduced myocardial infarct areas and induced anti-inflammatory response. Representative images and quantification of AAR and infarct size were at 3 d after I/R were measured by Evans blue and TTC (**a**). AAR was shown as percentage of the respective total heart areas and infarct size was expressed as percentage of AAR. Blue was unaffected heart muscle; red plus white was risk zone; white was infarcted tissue. LDH, cTnI, IL-10, IL-1β and TNF-α levels of mice blood serum after I/R were detected by a commercial kit at 3 d after I/R (**b**). Data are shown as Mean ± SEM, *n* = 5 per group. ^***^P < 0.001 *v*s Sham; ^#^*P* < 0.05, ^##^*P* < 0.01, ^###^*P* < 0.001 versus I/R; ^+^*P* < 0.05, ^++^*P* < 0.01 versus MSC

As shown in Fig. 6a, both MSCs and sevoflurane preconditioned MSCs significantly diminished infarct size compared with that in I/R group. This reduction of infarction in sevoflurane preconditioned MSCs group was further significant compared with that in MSCs (Fig. 6a). Similar changes were found in myocardial serum LDH, cTnI, IL-1β and TNF-α levels. LDH, cTnI, IL-1β and TNF-α levels were significantly augmented in I/R group, which were reversed by both MSCs and sevoflurane preconditioned MSCs (Fig. 6b). Serum IL-10 level was significantly increased under I/R compared with sham group, which further raised in both MSCs and sevoflurane preconditioned MSCs groups (Fig. 6b). All serum alterations described above were more significant in sevoflurane preconditioned MSCs group than in MSCs alone group (Fig. 6b). These results indicated that anti-inflammatory response is involved in the cardiac protection of sevoflurane preconditioned MSCs against I/R injury.

## Discussion

We found that sevoflurane preconditioning improves MSCs-induced angiogenesis under H/R via activating TRPC6-modulated HIF-1/CXCR4/VEGF axis and strengthens the cardioprotective effect of MSCs against I/R injury. Previous works including our own have shown that sevoflurane attenuates I/R injury in multiple organs including lung, brain and heart [4, 6, 8]. Sevoflurane preconditioning has been reported to produce protective effects on survival and migration of MSCs by augmenting HIF-1α and VEGF expressions [9], which is beneficial to boost angiogenesis and to improve the therapeutic efficacy of MSCs against cardiac I/R injury.

I/R injury is a result of the pathophysiologic stress, such as local and systemic inflammation, produced by an initial obstruction of blood flow to organ and the subsequent reperfusion [21]. The heart is susceptible to I/R and irreversible damages including myocardial necrosis and contractile dysfunction are easily produced only after 20 min’s ischemia [22]. MSCs have been reported to enhance angiogenesis, alleviate myocardial infarction and improve cardiac function in the I/R heart through paracrine effect of VEGF, stromal cell-derived factor-1 (SDF-1) and extracellular matrix protein into the surrounding infarct area [23, 24]. However, the decreased viability of transplanted MSCs owing to poor blood supply and over-accumulation of reactive oxygen species restricts its protective effect against I/R injury. In the present study, we found that sevoflurane preconditioning promoted the survival and preserved structural integrity of MSCs in hypoxic environment. Correspondingly, sevoflurane preconditioning also improved the angiogenic effect of MSCs under H/R. Furthermore, VEGF release from MSCs was significantly increased by sevoflurane preconditioning under H/R. These results indicate that sevoflurane preconditioning promotes MSCs to secrete VEGF and induce angiogenesis, which benefits to alleviate H/R injury.

VEGF, a well-known factor to promote mitogenesis, migration and survival in endothelial cells, actively contributes to angiogenesis under I/R injury [25]. The secretion of VEGF in HUVECs is significantly increased under hypoxia and is reduced by treatment with an inhibitor of HIF-1α [26]. Additionally, Ca^2+^ influx is necessary in endothelial cell adhesion, motility and angiogenesis [27], and blocking TRPC6, a Ca^2+^ influx channel, decreases VEGF secretion [12]. Such angiogenic effect of TRPC6 under H/R might be related to up-regulation of HIF-1α expression [28], which was confirmed by our study that silence of TRPC6 using siRNA reversed the up-regulation of TRPC6, HIF-1α, CXCR4 and VEGF in sevoflurane preconditioned MSCs under H/R. Moreover, our current work showed that sevoflurane preconditioned MSCs promoted tube formation under H/R and reduced myocardial infarct size and cardiac dysfunction in mice exposed to I/R insult. HIF-1α and CXCR4 have previously been reported to up-regulate VEGF and promote angiogenesis [29, 30]. SDF-1α/CXCR4, a definitive target of HIF-1α signaling, is also involved in cell migration and post-ischemic angiogenesis [31]. All together, these results suggest that TRPC6 channels are required for sevoflurane preconditioning to up-regulate HIF-1α, CXCR4 and VEGF expression in MSCs under H/R, which may promote the migration and angiogenesis of MSCs ultimately leading to the myocardial protection against I/R injury.

Transplantation of MSCs and sevoflurane preconditioned MSCs significantly improved EF and FS and decreased infarct size compared with those in I/R group after reperfusion for 3 d. They were consistent with the biomarkers of cardiomyocyte injury, serum LDH and cTnI. IL-10 levels were significantly augmented while IL-1β and TNF-α level were markedly decreased in serum from I/R mice transplanted with sevoflurane preconditioned MSCs. These results are consistent with previous studies [32, 33] and indicate that sevoflurane preconditioning advances MSCs to suppress inflammatory response which may help to prevent microvascular obstruction and relieve acute myocardial I/R injury.

## Conclusion

The current results suggest that sevoflurane preconditioning promotes MSCs to induce angiogenesis under H/R, which may be mediated by TRPC6-regulated HIF-1α/CXCR4/VEGF pathway and helps to relieve myocardial I/R injury.

## Supplementary Information

**Additional file 1. Fig. S1** The effect of sevoflurane preconditioning on Bcl-2 and Bax expression in MSCs under 12 h hypoxia and 2 h reoxygenation (H/R). M+O2, MSCs under normoxia; M-O2, MSCs under H/R; MS-O2, sevoflurane preconditioned MSCs under H/R. Data are shown as Mean ± SEM, n = 3 per group, *P<0.05, **P<0.01 vs M+O2, #P<0.05, ##P<0.01 vs M-O2.

## Data Availability

The data supporting the findings of the current study are available from the corresponding author on reasonable request.

## Abbreviations

MSCs: Mesenchymal stem cells
I/R: Ischemia/reperfusion
TRPC6: Transient receptor potential canonical channel 6
AV/PI: Annexin V/propidium iodide
HIF-1α: Hypoxia-inducible factor-1α
CXCR4: Chemokine receptor 4
VEGF: Vascular endothelial growth factor
H/R: Hypoxia and reoxygenation
siRNA: Small interfering RNA
HUVECs: Human umbilical vein endothelial cells
TTC: Triphenyltetrazolium chloride
TRP: Transient receptor potential
LAD: Left anterior descending
LV: Left ventricular
LVEDD: LV end-diastolic diameter
LVESD: End-systolic diameter
EF: Ejection fraction
FS: Fraction shortening
AAR: Area at risk
