# Supplementary material for: Sevoflurane preconditioning promotes mesenchymal stem cells to relieve myocardial ischemia/reperfusion injury via TRPC6-induced angiogenesis
Source: Stem Cell Res Ther. 2021 Nov 22;12:584. doi: 10.1186/s13287-021-02649-3 (PMC8607627; doi:10.1186/s13287-021-02649-3)
Supplement: Supplementary file 1 — Additional file 1. Fig. S1 The effect of sevoflurane preconditioning on Bcl-2 and Bax expression in MSCs under 12 h hypoxia and 2 h reoxygenation (H/R). M+O2, MSCs under normoxia; M-O2, MSCs under H/R; MS-O2, sevoflurane preconditioned MSCs under H/R. Data are shown as Mean ± SEM, n = 3 per group, *P<0.05, **P<0.01 vs M+O2, #P<0.05, ##P<0.01 vs M-O2. [file 13287_2021_2649_MOESM1_ESM.docx]

**Supplementary Material**

**Supplementary Figure**


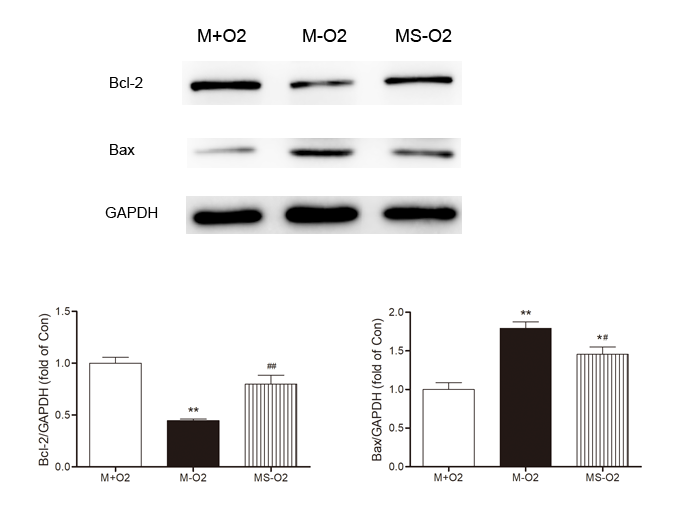


**Supplementary Fig. 1** The effect of sevoflurane preconditioning on Bcl-2 and Bax expressions in MSCs under 12 h hypoxia and 2 h reoxygenation (H/R). M+O2, MSCs under normoxia; M-O2, MSCs under H/R; MS-O2, sevoflurane preconditioned MSCs under H/R. Data are shown as Mean ± SEM, *n* = 3 per group, **P*<0.05, ***P*<0.01 *vs* M+O2, ^#^*P*<0.05, ^##^*P*<0.01 *vs* M-O2.
